# Dysentery

**Published:** 1889-07

**Authors:** A. Jacobi

**Affiliations:** President of the New York Academy of Medicine, Clinical Professor of the Diseases of Children in College of Physicians and Surgeons, New York, etc.


### Southern Medical Record.

A MONTHLY JOURNAL OF PRACTICAL MEDICINE.

☞ All communications must be addressed to R. C. WORD, Managing Editor.

"*Quicquid Præcipies Esto Brevis.*"

VOL. XIX ATLANTA, GA., JULY, 1889. NO. 7

### ORIGINAL AND SELECTED ARTICLES.

### DYSENTERY.

BY A. JACOBI, M. D.,

President of the New York Academy of Medicine, Clinical Professor of the Diseases of Children in College of Physicians and Surgeons, New York, etc.

The sufferings from dysentery are so intense, and the dangers from its acute or chronic state so threatening, that active measures must be taken at once. A brisk purgative ought to precede every other treatment. Castor oil in sufficient quantities, or calomel—according to age—in doses of from one to eight grains, will have a favorable effect, the latter acting both as a laxative and a disinfectant. The general rules of medicinal and hygienic treatment are those I laid down in my book on "The Intestinal Diseases of Infancy and Childhood" (G. S. Davis, Detroit, Michigan, 1889). It is these by which I shall be guided in most of the following remarks:

The food must be liquid. Milk and strained farinaceous decoctions must be the exclusive diet for the first acute stage. It is on the general condition of the patient that the administration of other articles of food, such as jellies, beef or mutton-broth, egg or alcoholic and medicinal stimulants (either general or cardiac), will depend in the course of the disease.

Great sensitiveness of the left hypogastric region and local heat will be alleviated by the application of ice. Very young infants, however, bear ice but a short time, whether applied to head or abdomen. I advise to watch the effect of the application either of the ice-bladder or the ice-cold cloth. Now and then, even in adults, we meet with an idiosyncratic incompatibility with cold. That has to be taken into account. Indeed, quite often warm applications of either water or poultices prove more efficient in regard to the two indications, which consist in alleviating irritation and reducing temperature.

The subnitrate and the subcarbonate of bismuth do not only cover and protect the mucous membrane, but have also a decided antifermentative effect. Thus bismuth is surely indicated in irritated conditions of the mucous membrane; it seldom fails when given in sufficient doses. There is no harm in sometimes giving it in such doses that part of the introduced material will pass through the entire length of the intestinal tract without undergoing decomposition. As its taste is not disagreable, it may be given together with tannin and opium; the daily dose ought not to be less than one drachm or a drachm and a half (4.0 to 6.0). At the same time the passages ought to be examined as to their reaction. Abundant acid, so frequently found in the slightest intestinal anomalies, requires the additional administration of alkalies. In most cases carbonate of lime is preferable to either magnesium or the carbonate or bicarbonate of sodium, the salts of both of which are apt to increase diarrhœa. Sometimes, particularly when the stomach can be relied upon, the salicylate of sodium may be added to the internal treatment. Beside the favorable effect of the sodium in the intestinal tract, the salicylic acid may prove beneficial both by its antifebrile and disinfectant action. Salol, one or two grains, or resorsin, one quarter or one-half a grain, may take its place. The latter is better tolerated than the former, but salol has a better chance to reach the lower part of the intestine.

Opium and its alkaloids are invaluable in the treatment of intestinal ulcerations. The objections to their use are decidedly exaggerated. Such accidents as have been reported in the journals as resulting from the administration of opium must be attributed to the fact that the dose was either absolutely or relatively too large, compared with the idiosyncrasy of the patient. Dysentery both requires and tolerates larger doses of opium than an average diarrhœa, no matter whether the latter be the result of catarrh or ulceration of the small intestine or the cæcum, or the upper part of the colon. In this respect dysentery stands abreast almost with peritonitis. The main indications are to relieve pain, reduce peristalsis and diminish the copious serous secretion: no other remedy fulfils all of them so well. For this purpose it ought to be given internally; for enemata containing opium may act favorably, but the more intense the tenesmus and the greater the hyperæmia, or the more extensive the ulceration, the less reliance can be placed in its effect, and the amount of the opiate thus brought into real action cannot be estimated. Among all the opiates I prefer a tincture, or the wine, or opium in substance, Dover's powder; but rarely have I injected morphia under the skin. The effect of the drug is easily watched and controlled by commencing with moderate doses, not repeating them too often, and being guided by the effect obtained. If opium is to be discarded, opium with hyoscyamus, or with belladonna, or hyoscyamus or belladonna alone, may take is place temporarily. Severe tenesmus may require the painting of the protruding part with Magendie's solution.

Astringents may either be given in combination with opium or separately, They are expected to pass wholly or partly through the entire length of the intestinal canal, thus coming into contact with the inflamed and ulcerous mucous membrane. Among those eligible are tannin, gallic acid and vegetables containing the same (ratanhia, catechu), besides subacetate of lead, nitrate of silver and pernitrate of rion.

The daily dose of tannin, when it is to be taken for a long time in succession, is from ten to fifteen grains, subacetate of lead, five to ten grains, nitrate of silver one-fourth to one-half grain. The latter ought not to be given more than a week or two in succession, for fear of argyria, two cases of which occurred in my own practice, and of my own making many years ago. All of these medicines are best taken, if possible, in the form of pills. They appear to be better tolerated, and are certainly more effective.

The use of keratin, when it becomes handier and cheaper, will facilitate their efficiency to a considerable extent.

Another antiseptic which I have frequently administered internally in every description of intestinal ulcerations, in both acute and chronic form, is naphthalin. For its doses, and the methods of its administration, and some account of its effect on intestinal ulceration in general, refer to the chapter on typhoid fever. We have to expect a great deal from such topical medication, and it appears that it will be one of the great refugees in all infectious diseases whose principal localization is in the intestine, as, for instance Asiatic cholera. With creolin I have no personal experience as yet.

Adults will take from fifteen to seventy-five grains daily, in powders, capsules or mucilage. Children bear, as a rule, according to their ages, from one-half of a grain to two or three grains, every two or three hours, in some mucilaginous substance. Some do not bear it well, but when such is the case the stomach will give warning at once.

The temperature will but rarely be so high as to require antipyretic medication. Frequent enemata will often reduce it effectively. Very young infants may demand an occasional dose of antipyrin or acetanilid (antifebrin) when the heat threatens either the nervous system or the normal structure of the tissues of the body.

Consecutive paralysis requires a mild galvanic current in the beginning. The daily application both to the spinal cord and the extremities need not exceed ten minutes; the electrodes must be large and the current reversed after five minutes. After a few weeks the interrupted current may be added the same length of time, but it must be applied to the paralyzed muscles only. Together with the latter, strychnia or (and) phosphorus may be used, in daily doses of one-thirtieth of a grain in the case of child of four or five years.

The local treatment of chronic dysenteric ulcerations requires the use of enemata. Their indications vary. They are to evacuate the bowels, or to reduce the irritability of the diseased intestine, or to accomplish an actual cure. These indications cannot be fulfilled separately; sometimes two, sometimes all three, can be at the same time. The nature and quantity and the temperature of the liquid to be injected depend, in part, on the end aimed at, in part on the irritability of the individual intestine. Sometimes the bowel objects to the introduction of small amounts; sometimes, however, large quantities are tolerated very easily indeed. To introduce small amounts, the selection of the syringe is a matter of indifference, provided the liquid enters the bowel gently and without pain. To inject large quantities, undue pressure and local irritation must be avoided. Thus the fountain syringe alone will answer; it ought to hang but a trifle above the level of the anus, say from six to twenty inches. The temperature of the liquid is not always a matter of great importance. Some recommend the injections to be ice-cold, some, however, tepid; both are frequently recommended as panaceas. But the practitioner will soon ascertain that some bear and require the one, some the other, some, indeed, very hot ones.

In my experience, for the large majority of patients tepid injections answer best. Not rarely is the intestine in such a condition of irritation that even small quantities of a very cold fluid are expelled at once. And again, there are cases in which enormous amounts of either cold or warm water are readily received. To accomplish the purpose of evacuating the bowel, plain water will often suffice, but three-fourths of one per cent. solutions of salt in water will usually prove more acceptable. Additions of bitartrate of potassa, or castor oil, have proved so uncomfortable in my cases that I have discarded them long ago. However, when the secretion of mucus on the rectal and intestinal mucous membranes was very large, one or two per cent. solutions of bicarbonate of sodium answered very well indeed. For the purpose of clearing the intestines, either of fæces or the morbid products, a single enema is sufficient. It ought to be repeated several times daily. When such mucus is secreted and tenesmus intense, it may be applied after every evacuation. In many cases the substitution of flaxseed tea or mucilage of gum acacia will prove advantageous. I have had to continue them weeks for both their evacuating and alleviating effect. When, however, the latter alone is aimed at—that is, when tenesmus is to be relieved—small quantities will usually suffice. An ounce or two of thin mucilage, or starch-water, or flaxseed tea, with tincture of opium, prove very comforting. Glycerin in water has been recommended for the same purpose. The former alone, or but slightly diluted, irritates, nay, cauterizes. It will require close judgment and individual experience to ascertain the degree of dilution, if it be used at all.

When a local curative effect is aimed at, injections of small quantities are sometimes insufficient. As the local lesions are often extensive, the amount to be injected must be pretty large. Almost always astringents are required. Sulphate of zinc or alumina, subacetate of lead, nitrate of silver, tannin, chlorate of potassium, ergotin, salicylic and carbolic acids and creosote have been recommended. Of the more common astringents I prefer alumina or tannin in one per cent. solutions. Creosote answered well in solutions one-half of one per cent. Salicylic acid resulted more frequently in pain than in benefit. Carbolic acid, in solutions of one-half of one per cent. has proved very beneficial, but I have learned long ago to be very careful in regard to its administration because of its poisonous effects, particularly in very young patients.

Injections of nitrate of silver may prove useful in cases not quite acute. Before the solutions of a quarter of one per cent., or of one or two per cent. are injected, the intestine ought to be washed out with warm water without salt. After the injection has been made it ought to be neutralized with a solution of chloride of sodium; it is still better to wash the anus and the portion of the rectum within easy reach with that solution before the medicinal injection be made. For even the mildest solutions, when acting on the sore sphincters are liable to give rise to intense tenesmus when no such care has been taken.

When the ulcerations are but few, or in the lower portion of the bowels only, small quantities suffice. But extensive lesions require large injections, the patient being on his side, or in the knee-elbow position. In these cases the nozzle syringe must be lengthened by attaching to it an elastic catheter, which is introduced as high up as possible, after the same plan that nutrient enemata are to be given. In a number of cases, both mild and severe, where neither the usual astringents nor nitrate of silver appeared to answer, I have been very successful these twenty years, when resorting to injections of subnitrate of bismuth. The drug is mixed with six or ten times its amount of water; of this mixture from one to three ounces (30.0 to 100.0) are injected into the bowel which has been washed out previously, twice or three times daily. The success was satisfactory, though a large portion of the injected mixture was soon expelled.

Suppositories containing the above substances may prove beneficial. But in order not to irritate they must be so soft as to melt readily. They may also contain some opium. But its admixture is not always sufficient to relieve the irritability of the rectum. Indeed, to accomplish this end opium must at least begin to liquify and to be absorbed, and absorption cannot be relied upon except where a part, at least, of the mucous surface is in a fair state of integrity. When no suppository can be tolerated, the painting with Magendie's solution, or the injection of a small quantity of olive oil with tincture of opium, may still be tried. The local application of cocaine relieves pain, but the drug is readily absorbed, and great caution must be used in its administration because of its poisonous effects.—*Archives of Pediatrics.*

